# Drug-related problems and its predictors among hospitalized heart failure patients at Jimma Medical Center, South West Ethiopia: prospective interventional study

**DOI:** 10.1186/s12872-022-02859-4

**Published:** 2022-09-19

**Authors:** Birbirsa Sefera, Mestawet Getachew, Yadeta Babu, Firomsa Bekele, Korinan Fanta

**Affiliations:** 1 Department of Pharmacy, College of Health Science, Mettu University, Mettu, Oromia Ethiopia; 2 School of Pharmacy, College of Public Health and Medical Sciences, Jimma University, Jimma, Oromia Ethiopia

**Keywords:** Drug therapy problems, Heart failure, Clinical pharmacist intervention

## Abstract

**Background:**

Drug-related problems are associated with high mortality, complications, prolonged hospital stay, compromised quality of life, and increased healthcare costs. This problem is high in patients hospitalized with chronic conditions such as heart failure. However, there are limited studies conducted on this area, particularly in Ethiopia.

**Objective:**

To evaluate drug-related problems, their predictors, and clinical pharmacist intervention among hospitalized heart failure patients at Jimma Medical Center, Ethiopia.

**Methods and participants:**

A prospective interventional study was conducted among hospitalized heart failure patients from September 30, 2020, to May 28, 2021, at Jimma Medical Center. Drug-related problems were sorted based on the Pharmaceutical Care Network Europe drug classification tool version 9.0. Patient's specific data were collected using a structured questionnaire. Data was analyzed using statistical software package version 23.0. Multivariate logistic regression analysis was used to identify independent predictors of drug-related problems occurrence and statistical significance was considered at a *p* value < 0.05.

**Results:**

A total of 237 heart failure patients were included in this study. The mean (SD) age was 49.06 + 17.79. About two-thirds (66.2%) of study patients had at least one drug-related problem during their hospital stay. A total of 283 drug-related problems were identified among 157 patients. Treatment effectiveness-related problem (55.48%) was the most common observed drug-related problem. The independent predictors of drug-related problems were khat chewing [AOR = 3.25, 95% CI = (1.46–7.23)], hospital stay > 18 days [AOR = 3.77, 95% CI = (1.93–7.37)]; presence of comorbid condition [AOR = 2.59, 95% CI = (1.35–4.96)] and polypharmacy [AOR = 2.94, 95% CI = (1.54–5.61)].

**Conclusion:**

The prevalence of drug-related problems was high among hospitalized heart failure patients in the study area. Chewing khat, prolonged hospital stay, comorbidity, and polypharmacy were the predictors of drug-related problems. Hence, to overcome these problems, clinical pharmacists, physicians, and other health professionals have to work in collaboration.

## Background

Heart failure (HF) is a condition in which a problem with the structure or function of the heart impairs its ability to supply sufficient blood flow to meet the body's needs [1]. HF emerges as a dominant form of cardiovascular disease in a developing country, this could be due to a switch toward a western lifestyle [2]. HF further increases hospitalization rates and, in turn, increases healthcare-associated costs [3]. Hence, the development of effective treatment regimens targeted at reducing the morbidity and mortality of heart failure patients has led to a large number of drugs with which heart failure patients are treated regularly. Increasing expenses for heart failure medications might prevent physicians from prescribing all the medications that are recommended in the recent guidelines. Although these regimens have beneficial effects in long-term treatment, the accumulation of side effects might prevent the patient from receiving full treatment [4]. Drug-related problems (DRPs) are a consequence of drug-related needs that have gone unmet. DRPs can occur for many reasons, such as inappropriate drug selection, inappropriate drug combination, or use of unproven medication instead of proven medication. The identification, resolution, and prevention of DRPs have been described as a core process of pharmaceutical care [5].

There are several classifications for DRPs. However, there is no single standardized classification in the world [6]. The PCNE classification system is commonly used and has better usability and internal consistency as it is updated and revised periodically. The current version is V9.0, which was developed during an expert workshop in February 2019. It is also meant to help health care professionals document DRP-information and to assess the nature, prevalence, and incidence of DRPs in the pharmaceutical care process. Moreover, unlike other DRPs classification systems, PCNE has different domains that are: problems domains, causes of drug-related problems, domains of planned intervention, domains for level of acceptance (of interventions) and domains for the status of the problem [7].

DRPs are relatively common in hospitalized patients and can result in patient morbidity and mortality as well as increased costs [6]. The estimated national prevalence of DRPs in Ethiopia was 70%. DRPs were caused by the presence of medical comorbidity, polypharmacy, significant drug interactions, and poor medication adherence [7]. On ambulatory HF patients at JUMC and TASH revealed that polypharmacy and the presence of comorbid conditions were the most common risk factors for DRPs, and treatment effectiveness related problems were the most common DRPs [8, 9]. DRPs contribute to a high number of morbidities and mortalities worldwide and are responsible for undesirable health consequences in patients that often result in hospitalization [8].

Studies revealed that one out of six patients admitted because of DRPs [9] and up to 30% of hospital admissions related to medication problems [10]. The costs associated with DRPs more than doubled, and hospital admissions became the primary contributor. DRPs are relatively common in hospital patients and can result in increased patient morbidity and mortality, thus increasing costs [2, 11]. From a retrospective review carried out in two hospitals on DRPs among cardiovascular disease (CVD) patients, 58.7% and 41.5% resulted in hospitalization [12].

DRPs among CVD patients were about three times more common in hospitalized patients than in outpatients [3]. Medication non-adherence is a major cause of hospitalization in patients with HF, which contributes enormously to health care costs. Thus, hospitalization is the primary contributor to the staggering medical cost of HF: $30.7 billion annually [13]. This cost is projected to increase more than twofold by 2030, making HF the most expensive condition billed to Medicare [14]. A study conducted at Felege Hiwot Referral Hospital (FHRH) in Bahirdar found that 96.1% of patients had at least one DRP [15]. At least two DRPs per patient were found at Tikur Anbessa Specialized Hospital (TASH) and Jimma Medical Center (JMC) at ambulatory clinics for HF patients [10, 11]. However, to the best of our knowledge, there has been limited study done on drug-related problems among hospitalized heart failure patients. This study is unique from previous research in that it was done on hospitalized heart failure patients; there was clinical pharmacist follow-up starting from the day of admission to discharge. Meanwhile, there was intervention on identified drug-related problems by a clinical pharmacist. Hence, the objective of this study was to identify drug-related problems and predictors in hospitalized heart failure patients and to evaluate clinical pharmacist intervention for treatment optimization.

Identifying and characterization of DRP among hospitalized heart failure patients is so crucial for healthcare professionals to optimize drug therapy that may influence health expenses, reduce morbidity and mortality, and increase quality of life. Drug-related morbidity and mortality can be reduced if patients are aware of drugs that pose a high risk for DRPs. It can also be used as an input in empowering pharmaceutical care services and promoting the significance of clinical pharmacists in the medical ward of the hospital. Finally, it will also be used as an input for further research.

## Methods and participants

### Study design and study setting

A hospital-based prospective interventional study was conducted from September 30, 2020 to May 28, 2021 in the medical ward of JMC, Oromia, Ethiopia. JMC is located in Jimma town, 352 km southwest of Addis Ababa, Ethiopia. It is among the largest teaching institutions in Ethiopia and is the only teaching and medical center hospital in the southwestern part of the country and provides services for the catchment population of about 20 million people.

### Study population and data collection procedure

All HF patients who were admitted to the medical ward at JMC were included in the study. Patients whose age was greater than 15 years old and those diagnosed with heart failure were included in the study. Patients who were not willing to participate and readmitted patients for whom data had been collected previously were excluded from the study. The data collectors trained for 2 days before starting data collection. Data was collected through medical record reviews of patients using a prepared standard checklist and structured questionnaire. The provisional diagnosis was taken after the patient stayed at least 24 h and confirmed by physicians. Drug-related problems were identified by evaluating the appropriateness of prescriptions regarding indication, dosage, and safety and by assessing patients. After data was collected, a clinical pharmacist reviewed the patients' therapy to assess DRPs. For the identified DRPs, interventions were provided immediately through discussion with individual prescribers. Additionally, recommendations were delivered during the round and the prescriber's acceptance was documented. DRPs that were not accepted were further discussed with senior physicians or residents for confirmation.

### Drug therapy problems identification and classification

Drug-related problems were identified by comparing patients' treatments with the updated clinical practice evidence-based guideline recommendations commonly practiced in a study area (AHA, ESTG) [1]. Patients' clinical characteristics were taken into account when deciding the appropriateness of the regimen. Then, a tool developed from the PCNE version 9.00 classification system for DRPs was used. The current PCNE-based classification of DRP has three primary domains for problems (P1-treatment effectiveness, P2-treatment safety, and P3-others). There are nine primary domains for causes (C1-drug selection, C2-drug form, C3-dose selection, C4-treatment duration, C5-dispensing, C6-drug use process, C7-patient-related, C8-patient transfer related, and C9-Others) and five primary domains for interventions (I1. no intervention, I2. at prescriber level, and I3. at patient level). I3. at the patient level, I4. at the drug level, and I5. elsewhere).

### Data quality assurance

The principal investigator trained and oriented data collectors for 2 days of study. The questionnaires were translated from English into Afan Oromo and Amharic, and then back-translated into English to assure their consistency. A pretest was conducted on 12 patients to check for uniformity and understandability of the checklist. The tool was modified after the results were obtained from the pretest. The principal investigator closely supervised the activity daily. At the end of each data collection day, the principal investigator checked the completeness of filled questionnaires.

### Data processing, analysis, and presentation

Data was entered into Epidata version 4.6.0.4 and exported to the Statistical Package for Social Sciences (SPSS) version 23 statistical analysis. First, the data was edited and checked for completeness and consistency. Then, it was exported into SPSS statistical analysis. Categorical variables were described by frequencies and percentages. Continuous variables were presented by means and standard. Univariate logistic regression was done to assess the association between the dependent and independent variables. Those variables with a *p* value 0.25 in univariate analysis were introduced into multivariate analysis. Variables with a *p* value of 0.05 were considered significant.

### Operational definition and definition of terms

*Drug-related problem* is an event or circumstance involving drug therapy that actually or potentially interferes with desired health outcomes.

*Hospitalized heart failure patients* heart failure is diagnosed clinically (using Framingham criteria) or confirmed with echocardiography. To make a clinical diagnosis, at least two major criteria or one major criterion and two minor criteria must be present at the same time. The following major criteria must be present: paroxysmal nocturnal dyspnea, neck vein distension, rales, radiographic cardiomegaly, acute pulmonary edema, third sound gallop, increased central venous pressure, and hepatojugular reflex. Minor criteria: bilateral ankle edema, nocturnal cough, dyspnea on ordinary exertion, hepatomegaly, and pleural effusion [16].

*Adverse drug reaction* is a noxious and unintended response to a drug that occurs at doses normally used for the prophylaxis, diagnosis, or treatment of disease that occurs during the study period [17].

*Polypharmacy* is defined as the concomitant use of five or more prescription medications [4].

*Clinical pharmacist intervention* is any action by a clinical pharmacist that directly results in a change in patient management or therapy.

*Comorbid condition* is the presence of another medical condition in addition to heart failure.

*Hospital stay* The time gap spent by the patient in the hospital from his/her admission till his/her discharge (the discharge date was determined by looking at his/her discharge date from his/her medical chart).

*Non-compliance* if the patient doesn't understand the instructions for drug taking or if the patient prefers not to take the medication; if the patient forgets to take the medication on time, if the drug product is too expensive for the patient, or if the drug product is not available [5].

*Insurance* Coverage of the cost of available medication provided by the health institution.

*Alcohol drinker* a person who has a history of drinking alcohol on a regular basis.

*Chewing khat* Those who had chewed Khat at some point in their lives [18].

*Inappropriate combination of drugs* is considered a drug-drug interaction (if risk D). So, within this study, it was taken as a treatment effectiveness-related problem or ADE (possibly) occurrence depending on the effect of drug one on the other drug.

## Results

### Socio-demographic characteristics of the study participants

Among 237 study participants included in this study, 122 (51.5%) were male and the mean age was 49.06 years ± 17.79. About 171(72.2%) of patients were residing in rural areas and more than half of patients were farmers. More than two-thirds (72.5%) of participants had no formal education (Table1).

**Table 1:** Socio-demographic characteristics among hospitalized heart failure patients at JMC

Socio-demographic characteristics and behavioral measures	Frequency (%)
Sex (male)	122 (51.5)
Age, years (mean ± SD)	49.06 ± 17.79
Age group	
< = 47	106 (44.7)
48–63	71 (30)
> = 64	60 (25.3)
Educational level	
No formal education	172 (72.5)
Primary education	51 (21.5)
Secondary education and above	14 (5.9)
Occupational status	
Unemployed	34 (14.3)
Farmer	152 (64.1)
Merchant	39 (16.5)
Government employee	12 (5.1)
Marital status	
Single	29 (12.2)
Married	171 (72.2)
Widowed or divorced	37 (15.6)
Residence	
Urban	66 (27.8)
Rural	171 (72.2)
Cost coverage method	
Insurance	71 (30)
Out of pocket	166 (70)
Social drug use	
Khat chewing	62 (26.2)
Alcohol drinking	31 (13.1)
Smoking	38 (16)

### Clinical characteristics of study participants

Of 237 patients included in the study, more than half of the patients had comorbidity 146 (61.6%). The most common causes of heart failure were IHD 94 (39.7%), followed by CRVHD 56 (23.6%) and CMP 51 (21.5%). About 56.1% of patients had stayed less than 18 days in the hospital, with a mean duration of 18.25 ± 8.82 (Table2).

**Table 2:** Clinical characteristics and laboratory investigation among hospitalized heart failure patients at JMC

Variables	Frequency (%)
Patient type	
Newly diagnosed HF patients	134 (56.5)
Known HF patients	103 (43.5)
NYHA Class	
II	13 (5.5)
III	58 (24.5)
IV	166 (70)
Etiology of heart failure	
IHD	94 (39.7)
CRVHD	56 (23.6)
CMP	51 (21.5)
HHD	23 (9.7)
Others*	13 (5.5)
Comorbid condition	146 (61.6)
Anemia	43 (29.5)
Atrial fibrillation	41 (28.1)
Hypertension	36 (24.7)
Chronic kidney disease	25 (17.1)
Acute kidney injury	24 (16.4)
Diabetes mellitus	21 (14.4)
Thrombosis	15 (10.3)
Others**	33 (22.6)
Number of comorbidity	
1	90 (61.6)
≥ 2	56 (38.4)
Hospital stay (mean ± SD)	18.3 ± 8.8
< 18	133 (56.1)
≥ 18	104 (43.9)
Serum electrolyte	
Potassium	
< 3.5 mmol/l	25 (12.8)
> 5.5 mmol/l	51 (26.2)
3.5–5.5 mmol/l	119 (61)
Sodium (< 135 mmol/l)	69 (35.4)
135–147 mmol/l	126 (64.6)
Renal function test	
Serum creatinine (mg/dl) (> 1.2)	69 (29.6)
(0.34–1.2)	126 (70.4)
Vital sign	
Systolic blood pressure (mmhg) (≥ 130)	33 (13.9)
Diastolic blood pressure (mmhg) (≥ 80)	29 (12.2)
Heart rate (bpm) (≥ 100)	46 (19.4)
(60–100)	191 (80.6)
Ejection fraction (%)	
≤ 40	104 (54.5)
41–49	23 (12)
≥ 50	64 (33.5)
Coagulation profile	
INR (< 2)	49 (85.9)
(2–3)	8 (14.1)
Prothrombin time (< 25)	50 (87.7)
(25–50)	7 (12.3)
Liver function test	
AST (≥ 40)	54 (26.1)
< 40	153 (73.9)
ALT (≥ 40)	43 (20.8)
< 40	164 (79.2)
Complete blood count	
White blood cell (< 4.5)	37 (16)
4.5–11	175 (75.8)
> 11	19 (8.2)
Hemoglobin, g/dl (≤ 8)	18 (7.8)
> 8	213 (92.2)
Platelet (< 150)	36 (15.6)
150–450	195 (84.4)

*IHD* ischemic heart disease, *CRVHD* chronic rheumatic valvular disease, *HHD* hypertensive heart disease, *CMP* cardiomyopathy, *NYHA* New York Heart Association, *INR* internationalized normal ratio, *AST* aspartate transaminase, *ALT* alanine transaminase

*Corpulmonale (2.1%), degenerative valvular disease (2.1%), and thyrocardiac disease (1.3%)

**Thyrocardiac disease (5.9%), chronic pulmonary disease (4.6%), HIV/AIDS (1.7%), tuberculosis (1.3%), and gout (0.4%)

### The prevalence, type, and causes of drug-related problems

**Fig. 1:**
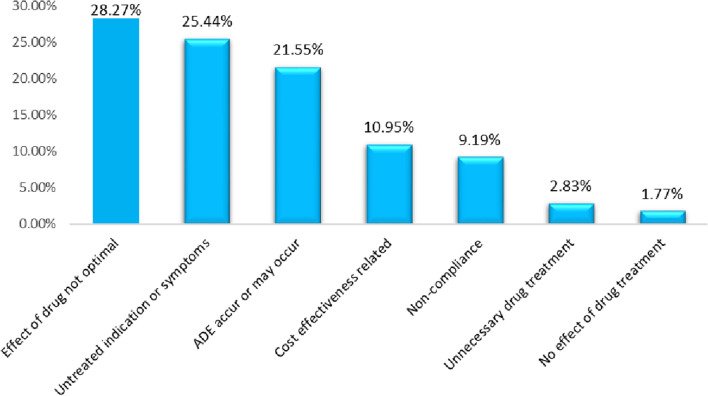
Types of DRPs among hospitalized heart failure patients at JMC from September 30, 2020, to May 28, 2021

Of a total of 237 patients, 157 (66.2%) patients experienced drug-related problems. During the study period, a total of 283 DRPs were identified. The average number of DRP per patient was 1.19 ± 1.18. Among patients who experienced DRPs, 80 (33.8%) had 1 DRP, 45 (18.9%) 2 DRPs and 32 (13.5%) ≥ 3 DRPs. The most commonly found DRPs were treatment effectiveness related (no effect of drug treatment, untreated indication, the effect of drug not optimal) 55.48%, followed by others (unnecessary drug treatment, compliance and cost-effectiveness related) 22.97% and safety-related (ADE occurs or may occur 21.55% (Fig. 1). Three hundred twenty-seven causes of DRPs were identified. Drug selection (33.33%), dose selection (20.49%), and patient-related were the most common causes (Table 3).

**Table 3:** Causes of DRPs among hospitalized heart failure patients at JMC from September 30, 2020, to May 28, 2021

Cause domain, total = 327	Frequency (%)
C1: Drug selection causes	109 (33.33)
New indication for drug treatment	67 (61.47)
Inappropriate drug according to guidelines	19 (17.43)
Contra-indicated	7 (6.42)
No indication for drug	6 (5.50)
Inappropriate combination of drugs, drugs, and foods	6 (5.50)
Inappropriate duplication of therapeutic agents	4 (3.68)
C2: Drug form causes	33 (10.09)
Inappropriate drug form	33 (100)
C3: Dose selection causes	67 (20.49)
Dosage regimen not too frequent	32 (47.76)
Drug dose too high	17 (25.37)
Drug dose too low	10 (14.93)
Dosage regimen too frequent	8 (11.94)
C4: treatment duration causes	1 (0.31)
Duration of treatment too long	1 (100)
C5: Dispensing causes	7 (2.14)
Prescribed drug not available	5 (71.43)
Necessary information not available	2 (28.57)
C6: Drug use process causes	17 (5.20)
Drug under administered	11 (64.70)
Inappropriate timing of administration	3 (17.65)
Drug not administered at all	3 (17.65)
C7: Patient-related causes	63 (19.27)
Patient unable to understand instructions	33 (52.38)
A patient takes less drug than prescribed	17 (26.98)
A patient takes more drugs than prescribed	7 (11.11)
Inappropriate timing or dosing intervals	5 (7.94)
A patient uses unnecessary drug	1 (1.59)
C8: Other causes	30 (9.17)
Not safe or drug-drug interaction	17 (56.67)
No or inappropriate outcome monitoring	13 (43.33)

### Drugs involved in drug-related problems

**Fig. 2:**
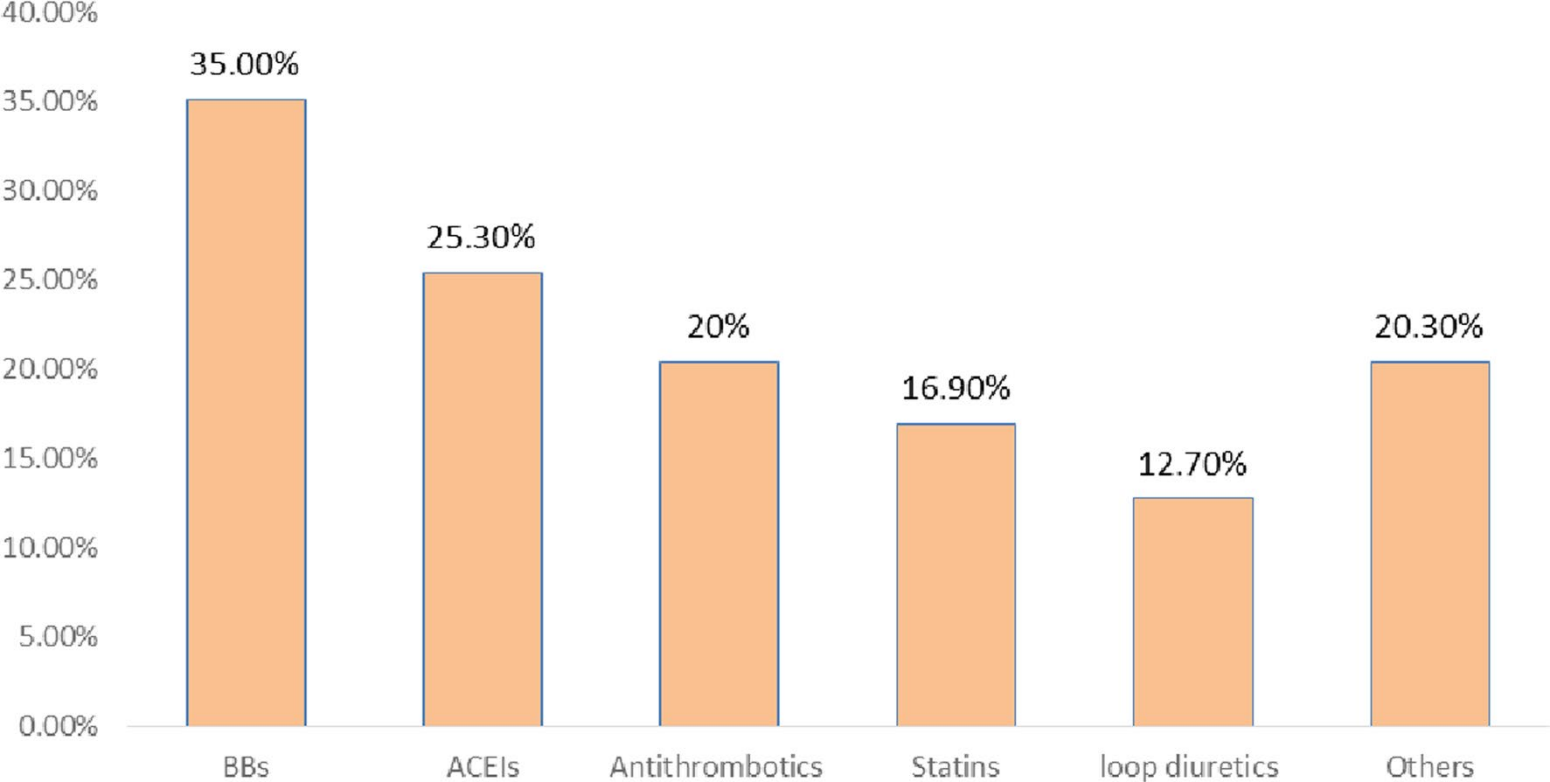
Common drug classes implicated in drug related problems among hospitalized heart failure patients at JMC from September 30, 2020 to May 28,2021. Others: Ferrous sulphate (4.6%), Spironolactone (3.8%), digoxin (2.1%), thionamides (2.1%), antibiotics (1.7%), calcium channel blockers (1.7%), omeprazole (1.3%), cimetidine (0.8%), amiodarone (0.8%), antiTB (0.8%) and hydrochlorothiazide (0.4%). *BBs* beta blockers, *ACEIs* angiotensin converting enzyme inhibitors

There were different classes of drugs involved among patients with drug-related problems. The most frequently encountered drug classes were beta-blockers (35%), of which 11% were unproven BBs. Angiotensin-converting enzyme inhibitors and antithrombotics were about 25% and 20% respectively. From the antithrombotic, anticoagulant was about six percent (Fig. 2).

### Intervention, acceptance rate, and outcome of intervention of drug-related problems

For the identified DRPs, a total of 408 interventions were delivered at different levels, out of this 38.73%intervention were done at the prescriber level, 93.04% of them were accepted. After an intervention, 72.08% and 18.37% of the problems were solved and not solved respectively (Table 4).

**Table 4:** Intervention, prescriber acceptance rate, and outcome of intervention for DRPs among heart failure patients at JMC from September 30, 2020, to May 28, 2021

	Frequency (%)
*Intervention domain* (N = 408)	
I1: Intervention at the prescriber level	158 (38.73)
The intervention proposed and discussed with the prescriber	133 (84.18)
Prescriber informed only	25 (15.82)
I2: intervention at the patient level	150 (36.76)
Patient drug counseling	80 (53.33)
Spoken to family member/caregiver	70 (46.67)
I3: Intervention at a drug level	100 (24.51)
Drug stopped	28 (28)
New drug started	27 (27)
Formulation changed	24 (24)
Drug changed	13 (13)
Instruction for use changed	6 (6)
Dosage changed	2 (2)
*Intervention acceptance domain* (N = 158)	
A1: Intervention accepted at the prescriber level	147 (93.04)
Intervention accepted and fully implemented	119 (80.95)
Intervention accepted and partially implemented	13 (8.84)
Intervention accepted but not implemented	10 (6.80)
Intervention accepted, implementation unknown	5 (3.41)
A2: Intervention not accepted	11 (6.96)
Not accepted; unknown reason	6 (54.55)
Not accepted; no agreement	5 (45.45)
*Problem status domain* (N = 283)	
O1: Problem solved	204 (72.08)
O2: Problem not solved	52 (18.37)
Lack of cooperation of prescriber	49 (94.23)
Lack of cooperation of the patient	3 (5.77)
O3: problem partially solved	18 (6.36)
O4: Problem status unknown	9 (3.19)

### Factors associated with drug-related problems

Univariate and multivariate logistic regression were carried out to determine predictors of DRPs. Univariate was done and for variables that had a p-value less than 0.25 multivariate logistic regression was done. The result of multivariate analysis of independent variables and DRPs revealed that history of khat chewing, presence of the comorbid condition, polypharmacy, and prolonged hospital stay greater than 18 days were significantly associated with DRPs (Table 5). The likelihood of having DRPs [AOR = 3.25, 95% CI = (1.46–7.23)] were about three times in patients who had a history of chewing khat as compared to those who had no history of khat chewing. It was found that patients who had polypharmacy were about three times more likely to have DRPs [AOR = 2.94, 95% CI = (1.54–5.61)] compared to those who had no polypharmacy. Similarly, patients with comorbidity were about three times more likely to have DRPs [AOR = 2.59, 95% CI = (1.35–4.96)] than without comorbidity. Patients who stayed more than 18 days in the hospital were four times more likely to have DRPs [AOR = 3.77, 95% CI = (1.93–7.37)] than those who stayed less than < 18 days in the hospital.

**Table 5:** Bivariate and multivariate analysis of independent factors associated with DRPs among hospitalized heart failure patients at JMC from September 30, 2020, to May 28, 2021

Variable	DRP status		COR	*p* value	AOR	*p* value
	Yes	No				
Sex (ref.female)	88 (56.1%)	34 (42.5%)	1.73 (1.00–2.97)	0.049	1.17 (0.56–2.45)	0.681
Age group						
≤ 47	72 (45.9%)	34 (42.5%)				
48–63	50 (31.8%)	21 (26.3%)	1.12 (0.59–2.16)	0.725	1.06 (0.49–2.29)	0.875
> 64	35 (22.3%)	25 (31.2%)	0.66 (0.34–1.27)	0.216	0.52 (0.24–1.12)	0.097
Residence (ref.no)	110 (70.1%)	61 (76.2%)	0.73 (0.39–1.35)	0.32		
Cost coverage method						
Insurance	49 (31.2%)	22 (27.5%)				
Out of pocket	108 (68.8%)	58 (72.5%)	0.84 (0.46–1.52)	0.56		
Khat chewing (ref.no)	50 (31.8%)	12 (15%)	2.65 (1.32–5.33)	0.006	3.25 (1.46–7.23)	**0.004**
Alcohol drinking (ref.no)	24 (15.3%)	7 (8.7%)	1.88 (0.77–4.58)	0.16	1.48 (0.49–4.41)	0.478
Cigarette smoking (ref.no)	28 (17.8%)	10 (12.5%)	1.52 (0.69–3.31)	0.29		
NYHA Class						
II	9 (5.7%)	4 (5%)				
III	36 (22.9%)	22 (27.5%)	0.73 (0.20–2.65)	0.63		
IV	112 (71.4%)	54 (67.5)	0.92 (0.27–3.13)	0.896		
Patient type						
Newly diagnosed HF	86 (54.8%)	48 (60)	0.81 (0.47–1.39)	0.443		
Known HF	71 (45.2%)	32 (40)				
Serum creatinine (> 1.2)	49 (31.2)	20 (25)	1.36 (0.74–2.50)	0.321		
AST						
> 40	38 (24.2%)	16 (20)	1.28 (0.66–2.47)	0.466		
ALT						
> 40	28 (17.8%)	15 (18.8%)	0.94 (0.46–1.91)	0.868		
Comorbid condition (ref.no)	114 (72.6%)	32 (40%)	3.98 (2.25–7.02)	< 0.001	2.59 (1.35–4.96)	**0.004**
Medication number						
< 5 medications	38 (24.2%)	48 (60%)				
≥ 5 medications	119 (75.8%)	32 (40)	4.69 (2.64–8.37)	< 0.001	2.94 (1.54–5.61)	**0.001**
Hospital stay						
< 18 days	72 (45.9)	61 (76.1)				
≥ 18 days	85 (54.1)	19 (23.7)	3.79 (2.07–6.93)	< 0.001	3.77 (1.93–7.37)	**< 0.001**

The predictors that are statistically significant in bold

## Discussion

Heart failure patients are more prone to DRPs due to different factors, like polypharmacy, comorbidity, and alteration in pharmacokinetic properties of HF patients, which result in impaired hepatic and renal blood flow [19]. Identifying, resolving, and preventing DRPs prevents detrimental health outcomes. Therefore, the identification and prevention of DRP occurrences are crucial.

The prevalence of DRP was found to be 66.2% and the average number of DRPs per patient was 1.19 + 1.18, which was lower than the study conducted at JUMC (83.5%) and 2.6 + 1.8. The difference could be due to setting differences; our study was conducted in hospitalized patients, in which senior physicians and clinical pharmacists are available more frequently than in ambulatory settings [10]. However, it is almost in line with studies conducted at TASH (65.5%) [11] and Gonder University Hospital (63.4%), or an average of 1.17 + 1.1 per patient [20]. Moreover, a study done on hospitalized heart failure patients at JUMC in 2014 showed DRPs were about 91% [21]. This difference from the current study could be due to CP intervention in current study.

The most common DRPs in our study were treatment effectiveness-related problems (55.48%) and the least was ADE occurrence (21.6%). Suboptimal drug treatment and untreated indications accounted for approximately 28% and 25% of treatment effectiveness issues, respectively. In contrast to this, a study conducted at the ambulatory clinic of JUMC in 2018 showed that treatment effectiveness was about 83%, of which suboptimal drug therapy and untreated indications were about 55% and 27%, respectively [11]. In addition to this, a study done in Barcelona showed that suboptimal drug therapy (31%) and the probability of ADE occurrence (16%) were comparable with our study [22]. Whereas, a study conducted at TASH showed that treatment effectiveness-related problems (39%) were lower than our findings [10]. Furthermore, a study done in the USA on outpatient heart failure showed that treatment effectiveness-related problems were about 36.8% [23]. The discrepancy could be due to differences in the study design and settings, clinical characteristics, population demographics, medication therapy used, methods of DRP identification and classification, or sample size difference. Non-compliance was about 9%, which was in line with studies done on ambulatory HF patients in JUMC (9%) and Barcelona, Spain (14%) [11, 22]. However, a study done at TASH showed that non-compliance was about 45% [10]. This could be due to differences in compliance assessment methods.

In our study, one-third of DRP causes were inappropriate drug selection and about 21% were dose selection-related problems. The new indication was about 60% of the causes of inappropriate drug selection, which was comparable with a study done at GUH which showed inappropriate drug selection and new indications were about 36% and 59%, respectively [20]. On the other hand, inappropriate drug selection (34% and dose selection, 27%), carried out at tertiary care teaching hospitals in southern India, was comparable with our findings [24]. However, a study on the general medical conditions of admitted geriatric patients at JUMC in 2017 showed that inappropriate drug selection was about 54% and the main causes of it were about 36% [25]. This may be due to different medical conditions and only the geriatric population.

Different classes of drugs were involved in DRPs among heart failure patients admitted to a medical ward. In the present study, the most common classes of drugs implicated in DRPs were BBs (35%) and ACEIs (25.3%), which was consistent with a study at the ambulatory clinic of JUMC, where BBs and ACEIs were 34.4% and 24.8%, respectively [11]. In addition to this, a study done in Taiwan showed that ACEI was about (21%) [26]. Moreover, studies conducted at the ambulatory clinic of TASH and in hospitalized HF patients at JUMC showed that BBs, ACEIs, and antithrombotics were the most commonly implicated drug classes in DRPs, likewise our findings [10, 21]. Finally, a study done on the detection and management of medication errors in internal wards at a teaching hospital in Iran revealed that cardiovascular medications were the class with the highest detected errors (31.6%) by clinical pharmacists [27].

The result of multivariate logistic regression showed that khat chewers, comorbidity, prolonged hospital stay, and polypharmacy were independent predictors of DRPs. According to the current study, patients with a social history of chewing khat have an independent effect on DRPs. To our knowledge, there has been no study that supports our findings. Somehow, a study conducted in southern India found that patients having a social history of alcoholism do have independent predictors of DRPs [24]. The plausible argument is that having a history of social drug use (chewing khat) may have contributed to patients' financial issues being disrupted. But still, more studies are needed to explicitly know the association between chewing khat and drug-related problems. In the current study, prolonged hospital stays were one of the independent predictors of DRPs among heart failure patients admitted to a medical ward. This was supported by studies done in Western Nepal and Pakistan [28] and the reason might be that the likelihood of getting multiple drugs increases with the increased length of hospital stay, which in turn will increase the likelihood of DRPs.

Comorbidity was another independent risk factor for DRPs in heart failure patients admitted to a medical ward. This is augmented by studies carried out at the ambulatory clinics of TASH and JUMC [10, 11, 29–33]. This could be due to patients with comorbidity being more likely to take more drugs to treat other diseases, causing disease-disease interaction, drug-drug interaction, and drug-disease interaction, which in turn makes patients more vulnerable to DRPs. Moreover, polypharmacy was also an independent predictor of DRPs, which was also supported by several studies [10, 11, 27, 30, 31, 34, 35]. This could be due to the fact that the more medications prescribed, the more drug-drug interactions, the risk of adverse events, difficulties with adherence, and the cost.

Clinical pharmacists' interventions in medical wards play a vital role in effectively identifying, resolving, and preventing DRPs. According to our study, clinical pharmacists' intervention acceptance rate was about 93%, of which about 81% of interventions were fully implemented and, from the outcome of the intervention, about 72% were solved. This result was comparable with studies carried out in Southern India and Karnataka, India, which revealed that clinical pharmacists' acceptance was about 97% and 96%, respectively [36, 37]. Moreover, a study carried out in Ghana, South Western Saudi Arabia, Northern Cyprus, and India showed that clinical pharmacists' intervention and acceptance rates were about two-thirds of the study population [38–40].

## Conclusion

Our study showed that the prevalence of drug-related problems was high in the medical ward of Jimma Medical Center. The most common identified DRPs were treatment effectiveness-related problems, which mainly include suboptimal effects of drugs and untreated indications. Chewing khat, staying in the hospital for an extended period of time, comorbidity, and polypharmacy were discovered to be independent predictors of drug-related problems. Clinical intervention acceptance and implementation rates were high, as was the intervention's solved outcome.

## Strength and limitations of study

We use PCNE as a tool that is validated, updated for researchers, and designed with separate codes for problems, causes, interventions, and outcome domains. Additionally, it is a prospective study, which increases the quality of the data. The strength of this study is that senior physicians and residents were included for confirmation if DRPs were not accepted by responsible physicians. This study has many limitations. Clinical pharmacist intervention may alter DRP prevalence. Cost reduction and the clinical impact of intervention are not studied. It doesn't classify the severity of DRPs into mild, moderate, and severe. Furthermore, the treatment outcome of drug-related problems was not addressed.

## Data Availability

Data used and analyzed during the current study are available from corresponding author on reasonable request.

## Abbreviations

ACEIs: Angiotensin-converting enzyme inhibitors
ADR: Adverse drug reaction
AHA: American Heart Association
ARB: Angiotensin receptor blocker
BBs: Beta-blockers
CP: Clinical pharmacist
CVD: Cardiovascular disease
DRP: Drug-related problems
HF: Heart failure
FHRH: Felege Hiwot Referral Hospital
HFSUH: Hiwot Fana Specialized University Hospital
ICU: Intensive care unit
JMC: Jimma Medical Center
MRA: Mineralocorticoid receptor antagonist
NSAID: Nonsteroidal anti-inflammatory drugs
PCNE: Pharmaceutical Care Network Europe
OR: Odds ratio
TASH: Tikur Anbessa Specialized Hospital
UK: United Kingdom
USA: United States of America

## References

[1] Seferovic PM, Ponikowski P, Anker SD, Bauersachs J, Chioncel O, Cleland JG (2019). Clinical practice update on heart failure 2019: pharmacotherapy, procedures, devices and patient management. An expert consensus meeting report of the Heart Failure Association of the European Society of Cardiology. Eur J Heart Fail.

[2] Savarese G, Lund LH (2017). Global public health burden of heart failure. Card Fail Rev.

[3] Ponikowski P, Anker SD, AlHabib KF, Cowie MR, Force TL, Hu S (2014). Heart failure: preventing disease and death worldwide. ESC Heart Fail.

[4] Beezer J, Al Hatrushi M, Kurdi A, Forsyth P (2021). Polypharmacy definition and prevalence in heart failure: a systematic review. Heart Fail Rev.

[5] Cipolle RJ, Strand LM, Morley PC (2012). Pharmaceutical care practice: the patient-centered approach to medication management.

[6] Basger BJ, Moles RJ, Chen TF (2015). Development of an aggregated system for classifying causes of drug-related problems. Ann Pharmacother.

[7] Schindler E, Richling I, Rose O (2021). Pharmaceutical Care Network Europe (PCNE) drug-related problem classification version 9.00: German translation and validation. Int J Clin Pharm.

[8] van den Bemt PM, Egberts TC, Brouwers JR (2000). Drug-related problems in hospitalised patients. Drug Saf.

[9] Knafl GJ, Riegel B (2014). What puts heart failure patients at risk for poor medication adherence?. Patient Prefer Adherence.

[10] Seid E, Engidawork E, Alebachew M, Mekonnen D, Berha AB (2020). Evaluation of drug therapy problems, medication adherence and treatment satisfaction among heart failure patients on follow-up at a tertiary care hospital in Ethiopia. PLoS ONE.

[11] Niriayo YL, Kumela K, Kassa TD, Angamo MT (2018). Drug therapy problems and contributing factors in the management of heart failure patients in Jimma University Specialized Hospital, Southwest Ethiopia. PLoS ONE.

[12] West LM, Williams JB, Faulkenberg KD (2019). The impact of pharmacist-based services across the spectrum of outpatient heart failure therapy. Curr Treat Options Cardiovasc Med.

[13] Viktil KK, Blix HS (2008). The impact of clinical pharmacists on drug-related problems and clinical outcomes. Basic Clin Pharmacol Toxicol.

[14] Adem F, Abdela J, Edessa D, Hagos B, Nigussie A, Mohammed MA (2021). Drug-related problems and associated factors in Ethiopia: a systematic review and meta-analysis. J Pharm Policy Pract.

[15] Tegegne GT, Gelaw BK, Defersha AD, Yimam B, Yesuf E (2014). Drug therapy problem among patients with cardiovascular diseases in Felege Hiwot referral Hospital, Northeast, Bahir Dar Ethiopia. IAJPR.

[16] Yancy CW, Jessup M, Bozkurt B, Butler J, Casey DE, Drazner MH (2013). 2013 ACCF/AHA guideline for the management of heart failure: executive summary: a report of the American College of Cardiology Foundation/American Heart Association Task Force on practice guidelines. Circulation.

[17] Rohilla A, Yadav S (2013). Adverse drug reactions: an overview. Int J Pharmacol Res.

[18] Sinshaw AE (2014). Prevalence and associated factors of khat chewing among Atse Fasil campus student in University of Gondar, North West Ethiopia. Malays J Med Biol Res.

[19] Udani SM, Koyner JL (2010). The effects of heart failure on renal function. Cardiol Clin.

[20] Abdela OA, Bhagavathula AS, Getachew H, Kelifa Y (2016). Risk factors for developing drug-related problems in patients with cardiovascular diseases attending Gondar University Hospital, Ethiopia. J Pharm Bioallied Sci.

[21] Wendie TF, Angamo MT (2020). Drug-therapy problems and predictors among hospitalized heart-failure patients: a prospective observational study. Drug Healthc Patient Saf.

[22] Gastelurrutia P, Benrimoj SI, Espejo J, Tuneu L, Mangues MA, Bayes-Genis A (2011). Negative clinical outcomes associated with drug-related problems in heart failure (HF) outpatients: impact of a pharmacist in a multidisciplinary HF clinic. J Cardiac Fail.

[23] Dempsey JT, Matta LS, Carter DM, Stevens CA, Stevenson LW, Desai AS (2017). Assessment of drug therapy-related issues in an outpatient heart failure population and the potential impact of pharmacist-driven intervention. J Pharm Pract.

[24] Gona OJ, Shambu SK, Madhan R (2021). Frequency and nature of drug-related problems in patients with acute coronary syndrome: role of the clinical pharmacist in coronary care practice. J Pharm Pract Res.

[25] Hailu BY, Berhe DF, Gudina EK, Gidey K, Getachew M (2020). Drug related problems in admitted geriatric patients: the impact of clinical pharmacist interventions. BMC Geriatr.

[26] Hsu W-T, Shen L-J, Lee C-M (2016). Drug-related problems vary with medication category and treatment duration in Taiwanese heart failure outpatients receiving case management. J Formos Med Assoc.

[27] Abbasinazari M, Talasaz AH, Eshraghi A, Sahraei Z (2013). Detection and management of medication errors in internal wards of a teaching hospital by clinical pharmacists. Acta Med Iran.

[28] Murtaza G, Khan MYG, Azhar S, Khan SA, Khan TM (2016). Assessment of potential drug–drug interactions and its associated factors in the hospitalized cardiac patients. Saudi Pharm J.

[29] Sharma S, Chhetri HP, Alam K (2014). A study of potential drug-drug interactions among hospitalized cardiac patients in a teaching hospital in Western Nepal. Indian J Pharmacol.

[30] Sewagegn N, Fekadu S, Chanie T (2015). Adherence to self-care behaviours and knowledge on treatment among heart failure patients in Ethiopia: the case of a tertiary teaching hospital. J Pharm Care Health Syst.

[31] Srikanth A (2017). Assessment of drug related problems and its associated factors among medical ward patients in university of Gondar teaching hospital, northwest Ethiopia: a prospective cross-sectional study. J Basic Clin Pharm.

[32] Tigabu BM, Daba D, Habte B (2014). Drug-related problems among medical ward patients in Jimma university specialized hospital, Southwest Ethiopia. J Res Pharm Pract.

[33] Mateti U, Rajakannan T, Nekkanti H, Rajesh V, Mallaysamy S, Ramachandran P (2011). Drug–drug interactions in hospitalized cardiac patients. J Young Pharm.

[34] Mohammed S, Poudel S, Laloo F, Madhur A, Robert R, Mathew B (2017). Assessment of drug-related problems in a tertiary care teaching hospital, India. Asian J Pharm Clin Res.

[35] Yusuff KB, Tayo F (2011). Frequency, types and severity of medication use-related problems among medical outpatients in Nigeria. Int J Clin Pharm.

[36] Celin A, Seuma J, Ramesh A (2012). Assessment of drug related problems in stroke patients admitted to a South Indian tertiary care teaching hospital. Indian J Pharm Pract.

[37] Shareef J, Sandeep B, Shastry C (2014). Assessment of drug related problems in patients with cardiovascular diseases in a tertiary care teaching hospital. J Pharm Care.

[38] Acheampong F, Nkansah FA, Anto BP (2016). Drug-related problems and their clinical interventions in a Ghanaian teaching hospital. Saf Health.

[39] Babelghaith SD, Wajid S, Alrabiah Z, Othiq MAM, Alghadeer S, Alhossan A (2020). Drug-related problems and pharmacist intervention at a general hospital in the Jazan Region, Saudi Arabia. Risk Manag Healthc Policy.

[40] Gökçekuş L, Mestrovic A, Basgut B (2016). Pharmacist intervention in drug-related problems for patients with cardiovascular diseases in selected community pharmacies in Northern Cyprus. Trop J Pharm Res.

